# Supplementary material for: Searching for Hypoglycemic Compounds from Brazilian Medicinal Plants Through UPLC-HRMS and Molecular Docking
Source: Plants (Basel). 2025 Nov 18;14(22):3517. doi: 10.3390/plants14223517 (PMC12656556; doi:10.3390/plants14223517)
Supplement: Supplementary file 1 [file plants-14-03517-s001.zip › plants-3944504-supplementary.pdf]

Figure S1. Dose-response curves determined for aqueous extracts from leaves of three Brazilian medicinal plants (concentration: 0.15625–10 mg/mL), and acarbose (concentration: 0.07813–10 mg/mL) in *α*-glucosidase inhibition assays. One-way ANOVA followed by Dunnett's multiple comparisons test revealed significant inhibition for *Justicia pectoralis* ($^{**}p < 0.01$) and *Amburana cearensis* ($^{*}p < 0.05$) compared to acarbose, while *Lippia origanoides* showed no significant difference ($^{ns}p > 0.05$).

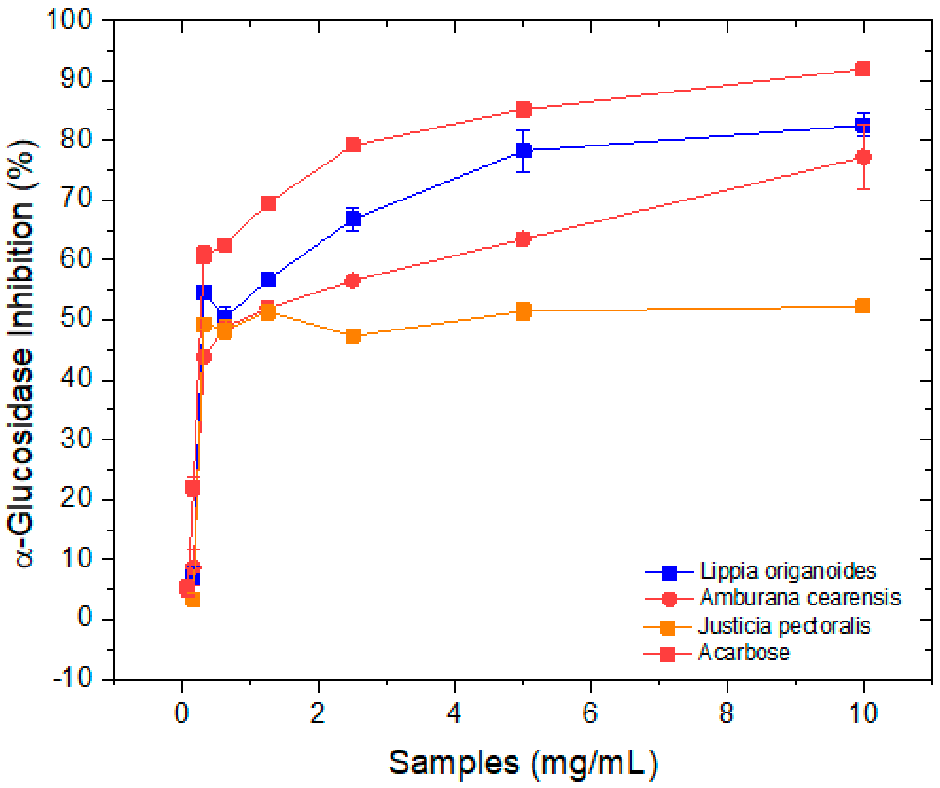

Figure S2. RMSD calculation for 2QMJ original structure and human α-glucosidase enzyme with hyperoside (quercetin-3-*O*-galactose).

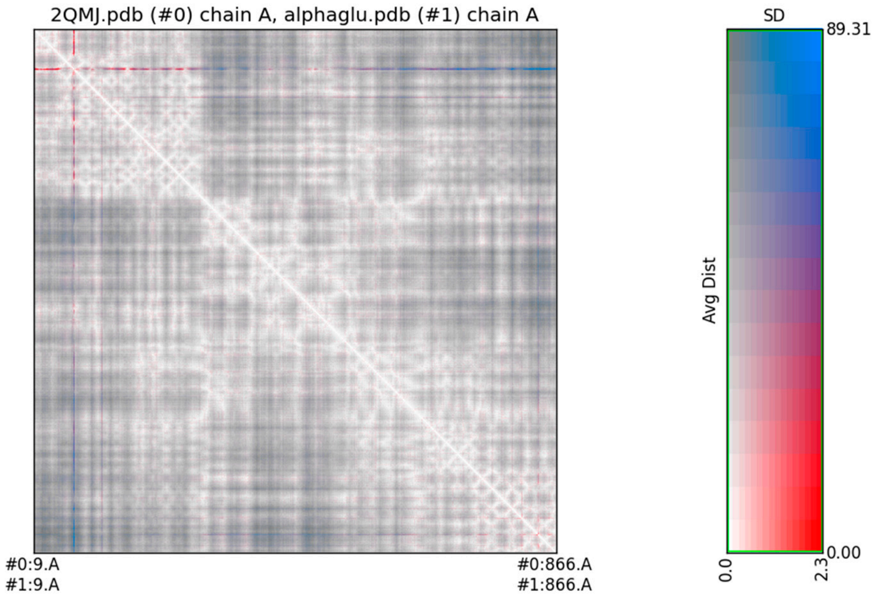

Figure S3. LigPlot diagram of trimethoxy-benzoic Acid, quercetin-3-O-galactose and quercetin-glucose complex with human α-glucosidase enzyme.

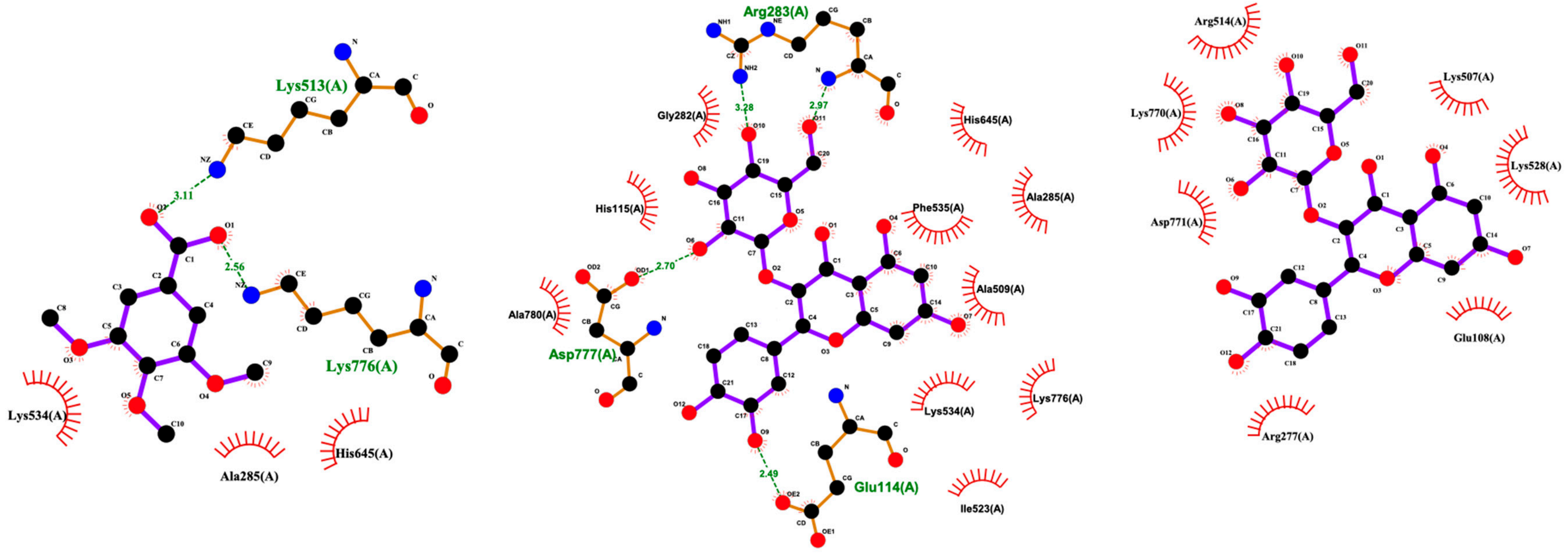

Trimethoxy Benzoic Acid **Quercetin_Galactose** **Quercetin_Glucose**

Figure S4: Dose-response curve for isoquercitrin, with an IC_{50} value of 0.08957 ± 0.00438 (concentration range: 0.00313–0.4 mg/mL) determined in an in vitro *α*-glucosidase inhibition assay.

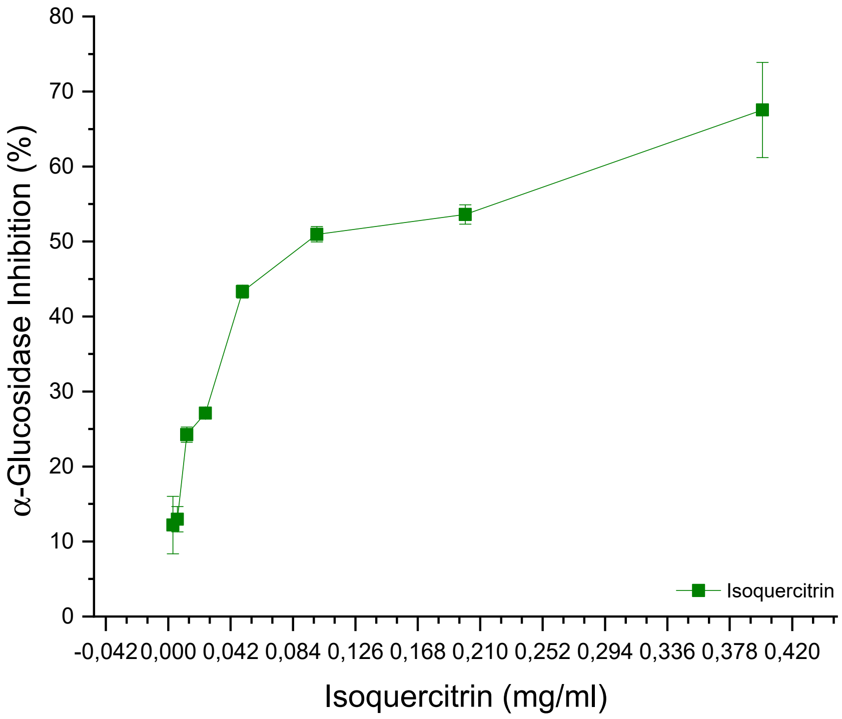

Table S1. Determination of 96-hour toxicological activity of aqueous leaf extracts from *Lippia origanoides, Amburana cearensis,* and *Justicia pectoralis* in adult zebrafish (*Danio rerio*).

Extract	Group and concentration (mg mL^{-1})					LC_{50} (mg mL^{-1}) CI
	C1 (3.125)	C2 (6.25)	C3 (12.50)	C4 (50.00)	C5 (100.00)	
Lippiao riganoides	0	0	1	0	2	>100 mg mL^{-1}
Amburana cearensis	0	0	0	0	0	>100 mg mL^{-1}
Justicia pectoralis	0	0	0	2	-	>50 mg mL^{-1}
Negative control (water)	0	0	0	0	0	-

LC_{50} = lethal concentration to kill 50% of individuals; CI = confidence interval.

ADME Properties Prediction

The pharmacokinetic profiles encompassing Absorption, Distribution, Metabolism, and Excretion (ADME) were predicted using the SwissADME web tool (http://www.swissadme.ch). The smiles files were uploaded directly to the server. The analyses were run using the default parameters for all compounds.

Table S2: ADME prediction for isoquercitrin (Quercetin-3-*O*-glucose), hyperoside (Quercetin-3-*O*-Galactose), Trimethoxy-benzoic acid and Acarbose.

**Property**	**Isoquercitrin**	**Hyperoside**	**Trimethoxy benzoic acid**	**Acarbose (Control)**
GI Absorption	Low	Low	High	Low
BBB Permeant	No	No	Yes	No
P-gp Substrate	No	No	No	Yes
CYP450 Inhibition	None	None	None	None
LipinskiViolations	2	2	0	3
Bioavailability Score	0.11	0.17	0.85	0.17
Water Solubility	Soluble	Soluble	Soluble	Highly Soluble
Synthetic Accessibility	High (5.28)	High (5.32)	Easy (1.83)	Very High (7.25)

Daina, A., Michielin, O. & Zoete, V. SwissADME: a free web tool to evaluate pharmacokinetics, drug-likeness and medicinal chemistry friendliness of small molecules. *Sci Rep* 7, 42717 (2017). https://doi.org/10.1038/srep4271

Table S3: Dunnet multiple comparison test for differences of mean percentage inhibition data for 95 % of confidence level.

Dunnett'smultiple comparisons test	MeanDiff,	95.00% CI of diff,	Significant?	Summary	Adjusted P Value	D		
Acarbose vs. Lippiaoriganoides	9.996	-6.092 to 26.08	No	ns	0.2928	A	*L. origanoides*	
Acarbose vs. Justicia pectoralis	24.84	8.750 to 40.93	Yes	**	0.0023	B	*J. pectoralis*	
Acarbose vs. Amburana cearensis	17.88	1.789 to 33.97	Yes	*	0.0276	C	*A. cearensis*	
**Test details**	**Mean 1**	**Mean 2**	**Mean Diff,**	**SE of diff,**	***n1***	***n2***	**q**	**DF**
Acarbose vs. Lippiaoriganoides	74.87	64.87	9.996	6.332	6	6	1.579	20
Acarbose vs. Justiciapectoralis	74.87	50.03	24.84	6.332	6	6	3.922	20
Acarbose vs. Amburana cearensis	74.87	56.99	17.88	6.332	6	6	2.823	20
